# Effects of eccentric contraction on force enhancement in rat fast‐twitch muscle

**DOI:** 10.14814/phy2.15797

**Published:** 2023-09-20

**Authors:** Jiayu Shi, Masanobu Wada

**Affiliations:** ^1^ Faculty of Sports Sciences Ningbo University Zhejiang China; ^2^ Graduate School of Humanities and Social Sciences Hiroshima University Hiroshima Japan

**Keywords:** force potentiation, molecular spring, passive force, sarcomere, titin

## Abstract

The aim of this study was to elucidate the effects of eccentric contraction (ECC) on force enhancement in rat fast‐twitch skeletal muscle. Gastrocnemius (GAS) muscles were subjected to 200 ECCs in situ by electrical stimulation. Immediately before and after the stimulation, isometric torque produced by ankle flexion was measured at an ankle angle of 90°. After the second torque measurement, the superficial regions of the muscles were dissected and subjected to biochemical and skinned fiber analysis. ECC did not induce changes in the amount of degraded titin. After ECC, isometric torques in the GAS muscles were markedly reduced, especially at low stimulation frequency. ECC increased passive torque in whole muscle and passive force in skinned fibers. Passive force enhancement and the ratio of passive force to the maximal Ca^2+^‐activated force, but not residual force enhancement, were augmented in the skinned fibers subjected to ECC. An ECC‐induced increase in titin‐based stiffness may contribute to the increased PFE. These results suggest that skeletal muscle is endowed with a force potentiation system that can attenuate ECC‐induced force reductions.

## 1 INTRODUCTION

The forces developed by striated muscles are divided into active and passive forces. Active force is generated by ATP‐driven interactions between myosin and actin filaments, whereas passive force is generated by elastic elements in stretched muscles. Titin is a giant protein with a molecular mass of 3–4 MDa that extends from the Z‐disk to the M‐band of the sarcomere. The I‐band region of titin contains flexible domains (immunoglobulin and PEVK domains), which are thought to contribute to passive force at long sarcomere lengths (SLs) (Freundt & Linke, 2019; Van der Pijl et al., 2019). At the muscle level, the connective tissue and titin are responsible for the passive force. In contrast, at the sarcomere level, most of the passive force is generated by titin (Freundt & Linke, 2019; Linke, 2018; Linke & Kruger, 2010).

Early evidence from rabbit skeletal muscle has suggested that a major role of titin is to maintain the myosin filament centered in the sarcomere during muscle contraction (Horowits et al., 1986). However, several studies performed over the last decades have provided data indicating that the passive and active force are tightly coupled to one another to generate the overall forces (Hessel et al., 2017; Linke, 2018). For instance, in skeletal and cardiac muscle, myofibrillar Ca^2+^ sensitivity is immediately increased by an elongation of the sarcomere (Konhilas et al., 2002; Lee et al., 2010; Patel et al., 2012; Shi et al., 2022). This phenomenon is referred to as length‐dependent activation (LDA), and in cardiac muscle, LDA has been demonstrated to be dependent on titin‐based passive force, with higher LDA in stiffer titin (Lee et al., 2010; Patel et al., 2012). Moreover, we have recently shown in rat skeletal muscle that the degree of an increase in the maximal force at a long SL is greater in single fibers with higher passive force than in those with lower passive force (Shi et al., 2021, 2022).

The other titin‐related force changes are two types of force enhancement (FE), that is, residual force enhancement (RFE) and passive force enhancement (PFE). RFE is defined as an increase in active isometric force of a muscle after active stretch, compared with the corresponding (same length and same activation) active isometric force of a muscle whose length is not changed during activation. PFE is defined as an increase in passive force of a muscle after active stretch, compared to the corresponding (same length) passive force of a muscle (Herzog, 2019). FEs have been demonstrated to increase with increasing the magnitude of stretch up to a certain sarcomere length (Hisey et al., 2009), indicating that the level of FE is dependent on titin‐based stiffness (Herzog, 2019).

Eccentric contraction (ECC) is a contraction, in which skeletal muscles are stretched while contracting. It occurs when a force applied to the muscle exceeds the instantaneous force generated by the muscle itself. In a recent study from our laboratory (Shi et al., 2022), immediately after ECC, titin‐based passive force was increased due to a decrease in phosphorylation by protein kinase A. Considering our results together with previous findings on FEs (Hisey et al., 2009), ECC may potentiate FEs. However, no studies have yet investigated these points.

These findings prompted us to investigate the effects of ECC on FEs. In this study, we tested the hypothesis that ECC, which increases titin‐based stiffness, potentiates both RFE and PFE. To this end, rat fast‐twitch muscles were subjected to ECC in situ and skinned fiber experiments were performed. The experiments conducted with gastrocnemius (GAS) muscles partially support our hypothesis.

## 2 MATERIALS AND METHODS

### 2.1 Ethical approval and animal care

Eight male Wistar rats (10–11‐week‐old) were used in this study (Charles River Laboratory, Japan). The animals were provided water and standard chow (CE‐2, CLEA Japan, Japan) ad libitum and housed in a thermally controlled room at 20–24°C with a 12:12‐h light–dark cycle. A mixture of medetomidine (0.4 mg⋅kg body wt^−1^), midazolam (2.0 mg⋅kg body wt^−1^), and butorphanol (2.5 mg⋅kg body wt^−1^) was intraperitoneally administered for anesthesia. At the end of the experiments, the rats were euthanized with an overdose of isoflurane, followed by cervical dislocation. All procedures performed were approved by the Animal Care Committee of Hiroshima University.

### 2.2 Eccentric contraction and isometric torque measurement

In preliminary experiments, we found that the isometric tetanic torque with surface electrodes increased with increasing voltage up to 40 V, but not above 40 V, and that the isometric tetanic torque induced by surface electrodes was similar to that induced by sciatic nerve stimulation. In our previous studies (Watanabe et al., 2015; Watanabe & Wada, 2016), tenotomy of the extensor digitorum longus, tibialis anterior, plantaris, and soleus muscles was performed before electrical stimulation to prevent these muscles from contributing to the torque produced by ankle flexion. In our preliminary experiments carried out later, we observed that the torque response of the hindlimb with tenotomy closely resembled that without tenotomy. These results suggest that (1) 50 V is a supramaximal voltage, (2) that most of motor units are recruited by electrical stimulation via surface electrodes, and (3) that the tenotomized muscles have little effect on torque production. Based on these results, surface electrodes and an electrical intensity of 50 V were used to stimulate GAS muscles, and tenotomy was not performed to minimize damage to the rats in this study.

Under anesthesia, the GAS muscles of the left hindlimb were electrically stimulated in situ. The left hindlimb was attached to a foot holder connected to a torque transducer (S‐21064, Takei, Japan). The knee and ankle were stabilized on the foot holder with a strap. The knee angle was maintained at 90° during the experiment. The muscle was stimulated every 4 s for 100 cycles using a 1‐s train of 1‐ms pulse at 80 Hz. During the stimulation, the foot was dorsiflexed by servomotor from 110° to 50° ankle angle at 60° s^−1^. This stimulation was repeated two times, and the sets were separated by a 5‐min recovery. Immediately before and after the ECC protocol, isometric torque, and passive torque in the left muscles were measured as follows:

- Step 1: The GAS muscle was stimulated at 20, 60, and 100 Hz for 1.5 s at 90° ankle angle. The contractions were separated by 1 min of rest.
- Step 2: After the measurement, whole muscle passive torques were measured at 80°, 70°, and 60° ankle angles. In this measurement, passive torque was defined as the torque level at 2 min after each ankle angle was set.

In steps 1 and 2, the torque at 90° ankle angle at rest was set to 0 mN·m. Muscles from the contralateral limb were used as rested controls to compare with muscles subjected to ECC (hereafter, referred to as “ECC muscles”) for amount of degraded titin, passive force in skinned fibers, RFE, and PFE. The rested and ECC muscles were excised after passive torque measurements. Mechanically skinned fibers were prepared from a part of the superficial region of the lateral muscle, and the superficial region of the medial muscle was stored at −80°C for biochemical analysis. The experiments were conducted in a room controlled at 25°C.

### 2.3 Degradation of titin

The GAS muscles (~50 mg) were homogenized in 9 vol. (vol.⋅mass^−1^) of an ice‐cold buffer containing 8 M urea, 2 M thiourea, 10 mM EGTA, 10 mM EDTA, 0.2 mM phenylmethanesulfonyl fluoride, 1.4 μM pepstatin A, 2.2 μM leupeptin, 0.83 mM benzamidine, and 0.13% 2‐mercaptoethanol (vol.⋅vol.^−1^). The protein content of the homogenate was determined using the Bradford assay with bovine serum albumin as the standard (Bradford, 1976). Forty micrograms of proteins were separated by agarose‐strengthened 2% SDS‐PAGE (Tatsumi & Hattori, 1995). Protein bands were visualized with Coomassie Blue R staining and analyzed using the ImageJ software. The proportion of degraded form to total titin was calculated.

### 2.4 Skinned fiber experiments

#### 2.4.1 Solutions and skinned fiber preparation

The maximal Ca^2+^ activation (max Ca^2+^) solution consisted of (in mM) 36 Na^+^, 126 K^+^, 90 HEPES, 8 ATP_total_, 10 creatine phosphate, 1 Mg^2+^_free_, 49.5 Ca‐EGTA, and 0.5 EGTA_free_ and had a pH of 7.09–7.11 (pCa = ∼4.7). The relaxation solution was similar to the max Ca^2+^ solution, but with 49.5 mM Ca‐EGTA replaced by 49.5 mM EGTA (pCa >9). Mechanically skinned fibers were prepared from the excised GAS muscle as previously described in detail (Lamb & Stephenson, 2018; Watanabe et al., 2015). Skinned fiber preparation was performed simultaneously by two different researchers. The skinned fibers obtained by one and the other researcher were used for measurements of passive force and FEs, respectively. These measurements were started within 15 min of muscle excision. All skinned fiber experiments were performed at room temperature (~25°C).

#### 2.4.2 Measurements of passive force

Passive force was defined as the steady‐state force level attained after stretching the fiber to a certain SL, or in the case of long SLs, as the force level reached 2 min after the stretch (Shi et al., 2021; Watanabe, Lamboley, & Lamb, 2019). The skinned fiber segment was attached to a force transducer (Muscle tester, SI, Germany) with a suture. The other end of the segment was secured with fixed forceps connected to a manipulator. The fiber was then placed in a polystyrene spectrophotometric vial containing the relaxation solution. SL was determined from the diffraction pattern produced by a He‐Ne laser beam passing through the skinned fiber (Stephenson & Williams, 1981). The SL was adjusted to 2.4 μm after 5 min of equilibration. The fiber was slacked off at 2.4‐μm SL and the passive force at this SL was 0 mN. The passive forces were measured at 2.6‐μm, 3.0‐μm, 3.4‐μm, and 3.6‐μm SLs (Shi et al., 2021, 2022). A digital microscope (×200 magnification) was used to determine the fiber diameter. The cross‐sectional area was calculated based on the average diameter measured at three different positions, assuming a cylindrical shape of the fiber. The passive force was normalized to the cross‐sectional area.

#### 2.4.3 Measurements of residual force and passive force enhancement

**FIGURE 1:**
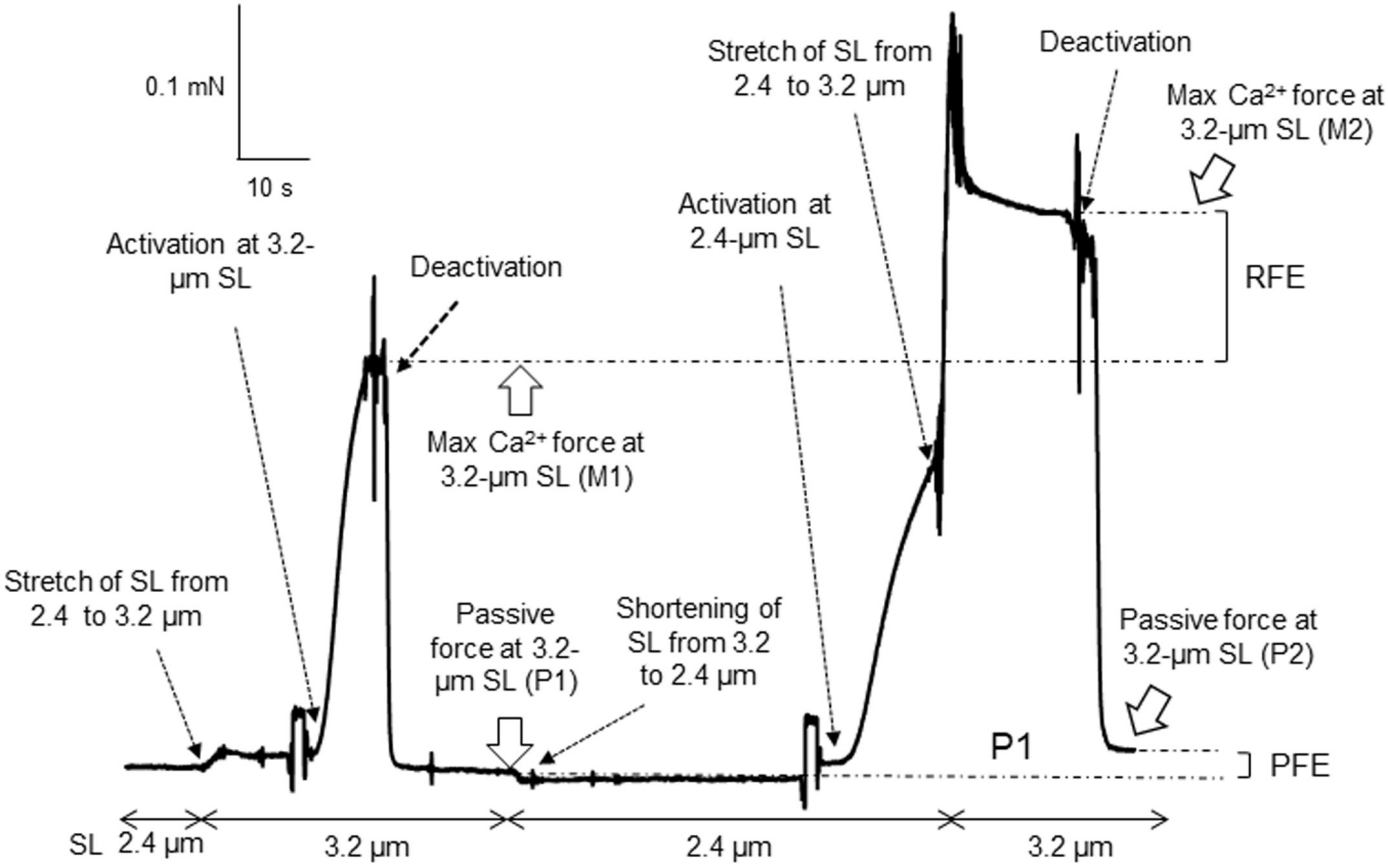
Force record in measurements of passive force enhancement (PFE) and residual force enhancement (RFE). Maximal Ca^2+^‐activated force (max Ca^2+^ force or M) and passive force (P) were evaluated using two different procedures. In the first procedure, the force (M1) was measured by immersing the skinned fiber to the maximal Ca^2+^‐activated solution after stretching the sarcomere length (SL) from 2.4 to 3.2 μm in the relaxation solution. P1 is the passive force at 3.2‐μm SL after M1 measurement. In the second procedure, the SL was stretched from 2.4 to 3.2 μm while the fiber was immersed into the maximal Ca^2+^‐activated solution (the max Ca^2+^ force measured is referred to as M2). P2 is the passive force at 3.2‐μm SL after M2 measurement. RFE is a difference between M1 and M2, and PFE is a difference in between P1 and P2.

To evaluate RFE and PFE, the skinned fibers were treated as follows (Figure 1).

- Step 1: The fiber was placed in the relaxation solution and slacked off at 2.4‐μm SL.
- Step 2: The SL was stretched to 3.2 μm.
- Step 3: The fiber was immersed into the max Ca^2+^ solution (activation).
- Step 4: After reaching the maximal force, the fiber was immersed into the relaxation solution (deactivation). The maximal Ca^2+^‐activated force (max Ca^2+^ force) and the passive force after deactivation in step 4 were termed maximal force (M) 1 and passive force (P) 1, respectively.
- Step 5: The SL was shortened to 2.4 μm.
- Step 6: The fiber was immersed in the max Ca^2+^ solution.
- Step 7: The SL was stretched to 3.2 μm in the max Ca^2+^ solution. The max Ca^2+^ force in step 7 was termed M2.
- Step 8: The fiber was immersed in the relaxation solution 20 s after stretching. The passive force after deactivation in step 8 was termed P2.

In step 6, the diffraction pattern produced by the laser beam sometimes disappeared or became blurred. In this case, the fiber was replaced in the relaxation solution and a fiber length difference in between 2.4‐μm and 3.2‐μm SL was measured in each fiber, using the manipulator. Based on the fiber length difference, the SL of the activating fiber was then adjusted to 3.2 μm. Our preliminary experiment demonstrated that excessively long immersion (more than ∼40 s) to the max Ca^2+^ solution often resulted in broken skinned fibers. M2 was then defined as the force level reached 20 s after the active stretch. Forces were normalized to the cross‐sectional area. The differences between M1 and M2 and between P1 and P2 are termed RFE and PFE, respectively.

### 2.5 Statistical analyses

Data are presented as mean + SD. Two‐way ANOVA, two‐way repeated ANOVA, Student's *t*‐test, or Student's paired *t*‐test were used to determine the effects of ECC, stimulation frequency, ankle angle, SL, and/or active stretch as appropriate (SigmaPlot 14.5 software, HULINKS). The Holm–Sidak method was used for post hoc analysis when significant differences were detected in the two‐way repeated ANOVA. The acceptable level of significance was set at *p* < 0.05 (a two‐tailed *p*‐value).

## 3 RESULTS

### 3.1 Isometric torque in whole muscle

**FIGURE 2:**
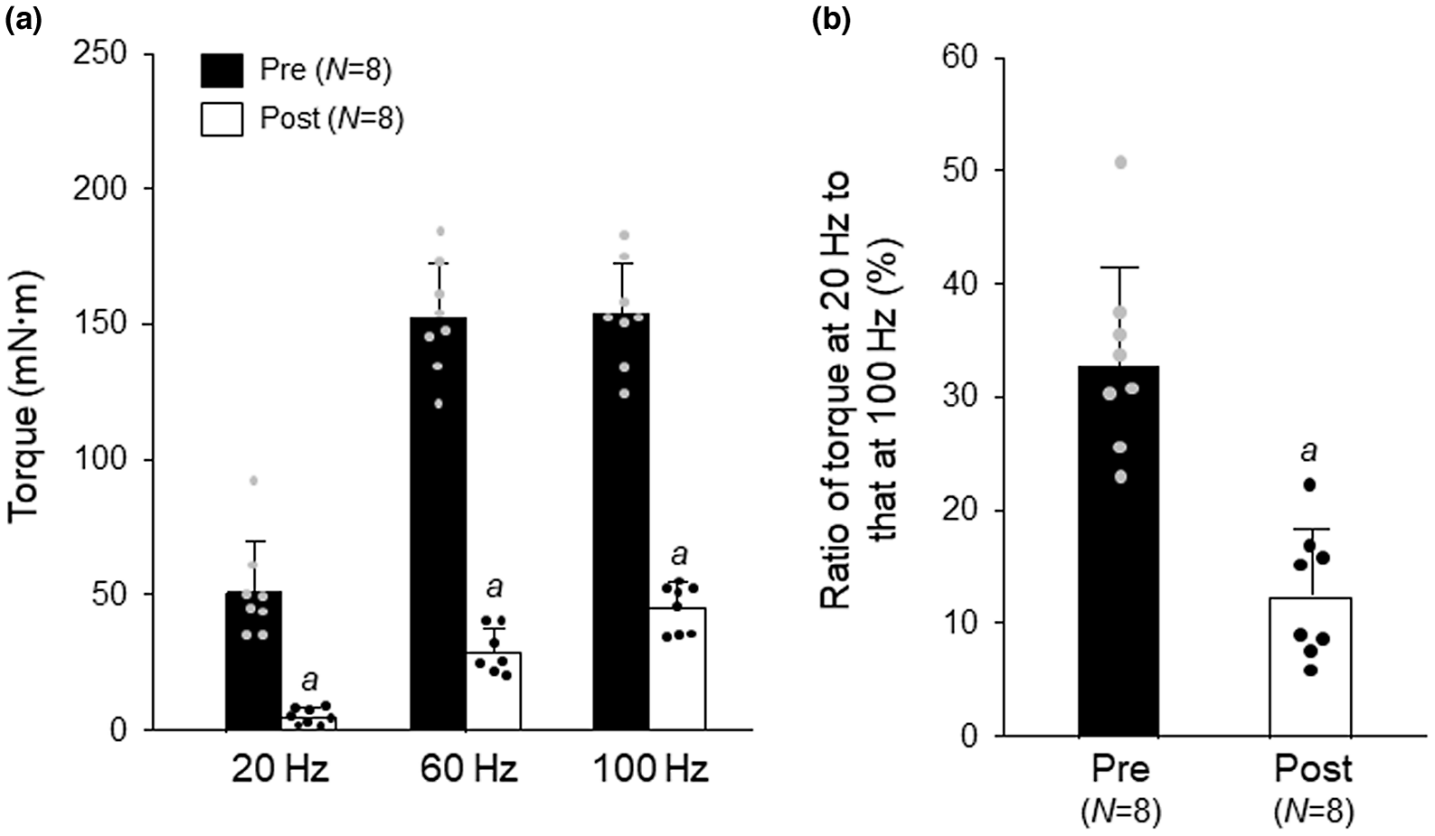
Effects of eccentric contraction (ECC) on isometric torque in whole muscle. Intact gastrocnemius muscle was electrically stimulated in situ at 80 Hz for 1 s every 4 s for 200 cycles. During the stimulation, the foot was forcibly dorsiflexed by a servomotor from 110° to 50° at 60°/s. (A) Isometric torque at pre‐ and post‐ECC. (B) Ratio of force at 20 Hz to that at 100 Hz. Values are means + SD; *N* denotes number of muscles. Individual data are displayed as gray or black circles. ^*a*^ *p* <0.05, vs. pre‐ECC (two‐way repeated ANOVA and paired *t*‐test for (A and B), respectively). Symbols for significant differences between stimulation frequencies are not shown. Post, post‐ECC; Pre, pre‐ECC.

There was a significant interaction between ECC and stimulation frequencies for isometric torque (Figure 2A). ECC induced a marked reduction in torque, which was greater at the lower frequencies. As a result, the low/high torque ratio (20 vs. 100 Hz) decreased at post‐ECC (Figure 2B). This phenomenon is referred to as “low‐frequency fatigue” and is consistent with our previous observation (Kanzaki et al., 2014).

### 3.2 Degraded titin

**FIGURE 3:**
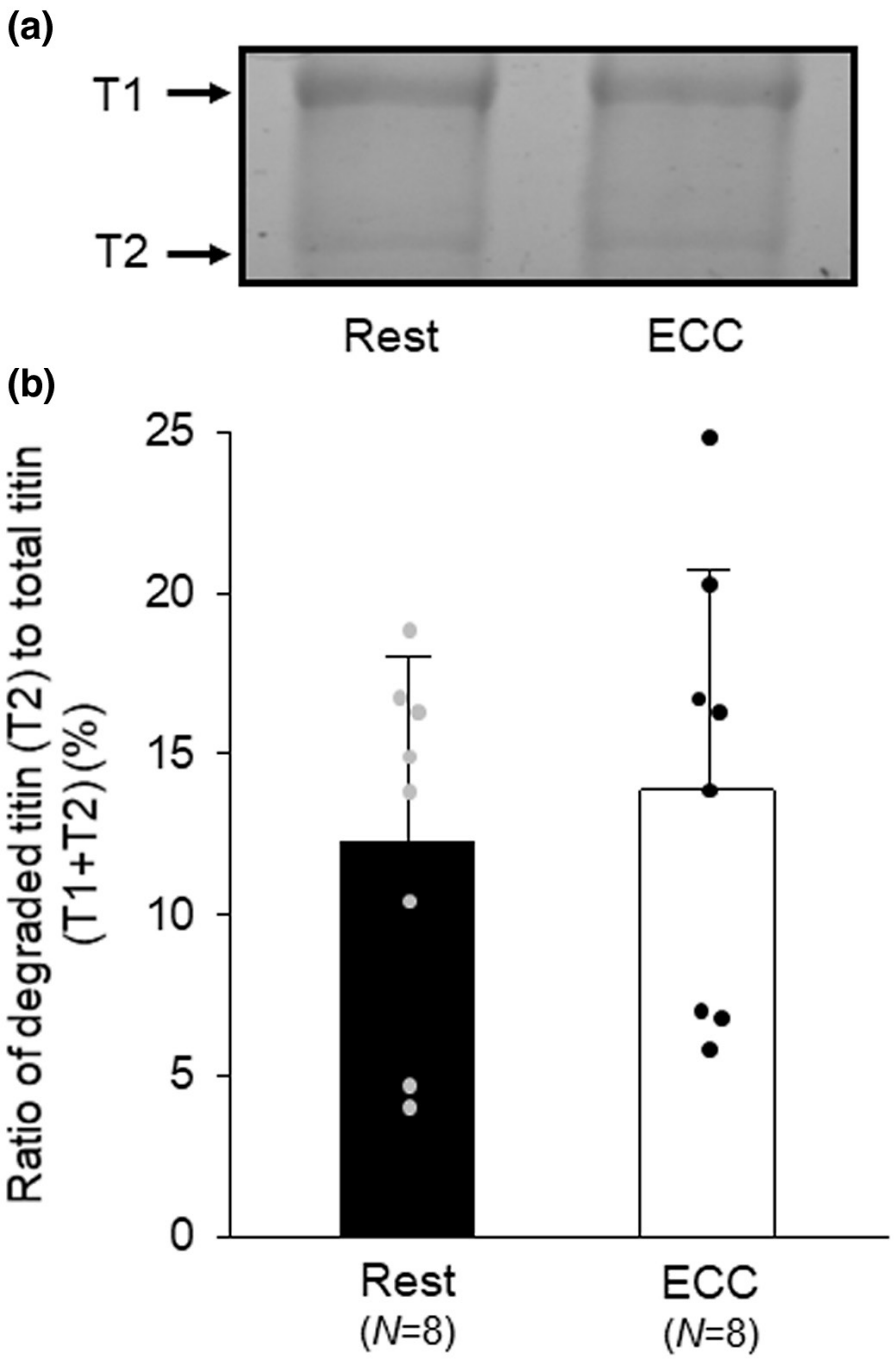
Effects of eccentric contraction on titin degradation. For the protocols of eccentric contraction (ECC), see legend of Figure 2. (A) Electrophoretic analysis of titin. Proteins were separated by agarose‐strengthened 2% SDS‐PAGE and were visualized with Coomassie Blue R staining. T1 and T2 are full‐length and degraded titin, respectively. (B) Ratio of degraded titin to total titin (T1 + T2). Values are means + SD; *N* denotes number of muscles. Individual data are displayed as gray or black circles.

Titin has been identified in vitro as one of the substrates of calpain‐3 (a skeletal muscle‐specific, Ca^2+^‐regulated protease) (Goll et al., 2003). Considering that calpain‐3 is activated after ECC (Kanzaki et al., 2014), ECC may result in titin cleavage. However, as presented in Figure 3, the amount of degraded titin was unchanged, a result consistent with our previous findings (Shi et al., 2022).

### 3.3 Passive torque in whole muscle and passive force in skinned fiber

**FIGURE 4:**
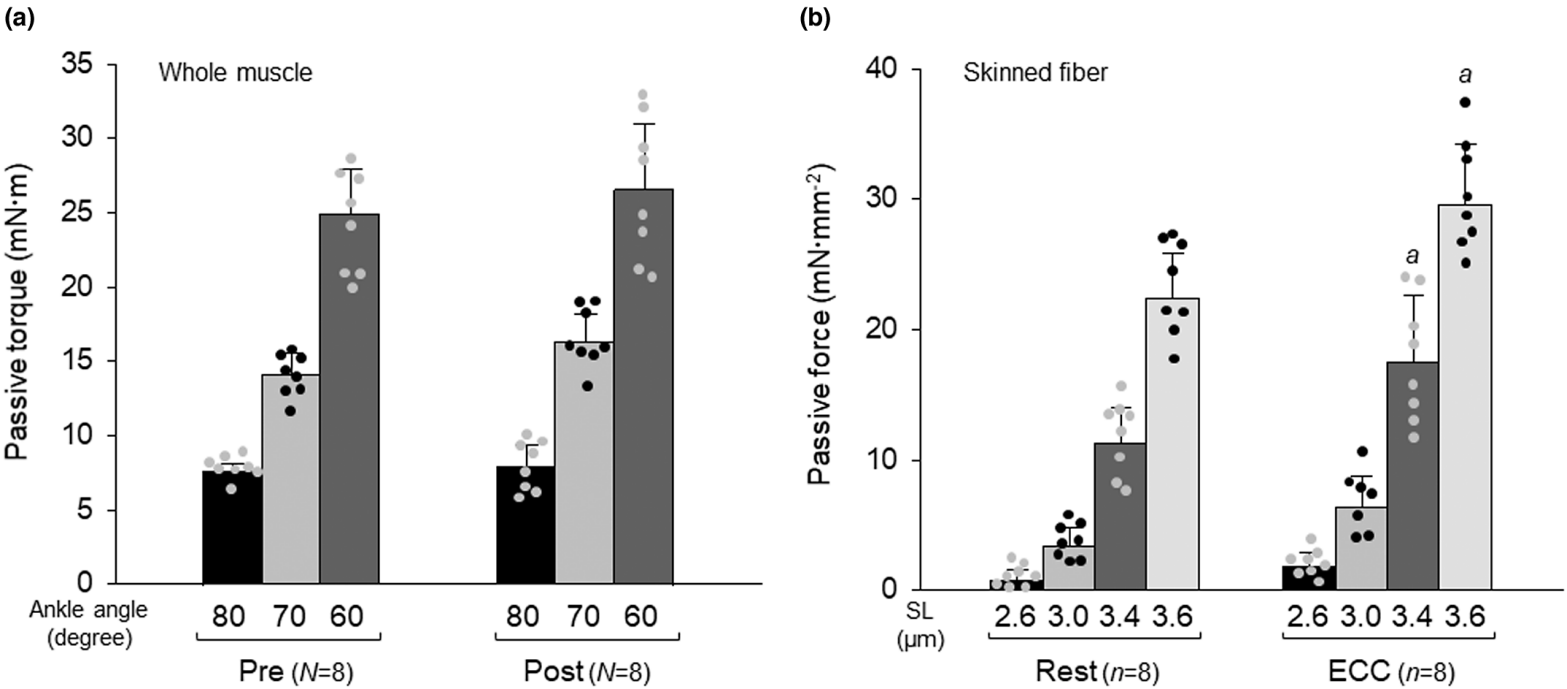
Effects of eccentric contraction on passive torque in whole muscle (A) and passive force in skinned fiber (B). For the protocols of eccentric contraction (ECC), see legend of Figure 2. Values are means ± SD; *N* and *n* denote number of muscles and skinned fibers, respectively. Individual data are displayed as gray or black circles. ^*a*^ *p* <0.05, vs. rested fibers in matching condition (two‐way ANOVA). A main effect of ECC was observed for whole muscle passive force (Pre < Post; two‐way repeated ANOVA). Symbols for significant differences between ankle angles and sarcomere lengths (SLs) are not shown in (A and B), respectively. ECC, fibers subjected to ECC; post, post‐ECC; Pre, pre‐ECC; Rest, rested fibers.

There was no interaction between ECC and ankle angle for passive torque (Figure 4A). The main effect was that the passive torque was higher in ECC than in rested muscles. A significant interaction between ECC and SL was observed for skinned fiber passive force (Figure 4B). The passive force at 3.4‐μm and 3.6‐μm SLs in fibers subjected to ECC (hereafter, referred to as “ECC fibers”) increased to a level of 155.8% and 132.6% of that in rested fibers, respectively.

### 3.4 Residual force enhancement in skinned fiber

**FIGURE 5:**
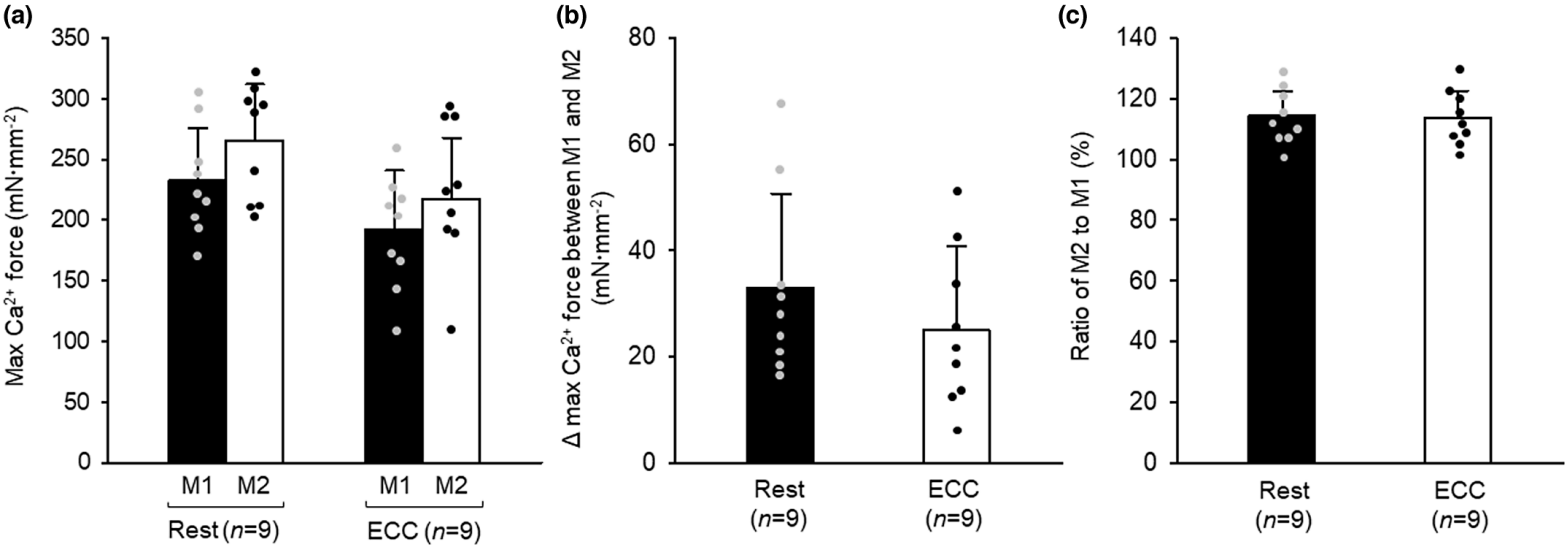
Effects of eccentric contraction and active stretch on maximal Ca^2+^‐activated force (max Ca^2+^ force) in skinned fiber. For the protocols of max Ca^2+^ force measurement and eccentric contraction (ECC), see legend of Figures 1 and 2, respectively. (A) max Ca^2+^ force (M) 1 and 2. (B) Difference between M1 and M2. (C) Ratio of M2 to M1. Values are means + SD; *n* denotes number of fibers. Individual data are displayed as gray or black circles. Main effects of ECC and active stretch were observed (Rest > ECC and M2 > M1; two‐way repeated ANOVA). Rest, rested fibers; ECC, fibers subjected to ECC.

There was no interaction between ECC and active stretch of SL for the max Ca^2+^ force (Figure 5A). The main effects were that max Ca^2+^ forces (M1 and 2) were lower in ECC than in rested fibers and that M2 was greater than M1. No differences in Δ max Ca^2+^ force (Figure 5B) or a percent increase (ratio of M2 to M1; Figure 5C) were observed between rested and ECC fibers, indicating that ECC does not potentiate RFE.

### 3.5 Passive force enhancement in skinned fiber

**FIGURE 6:**
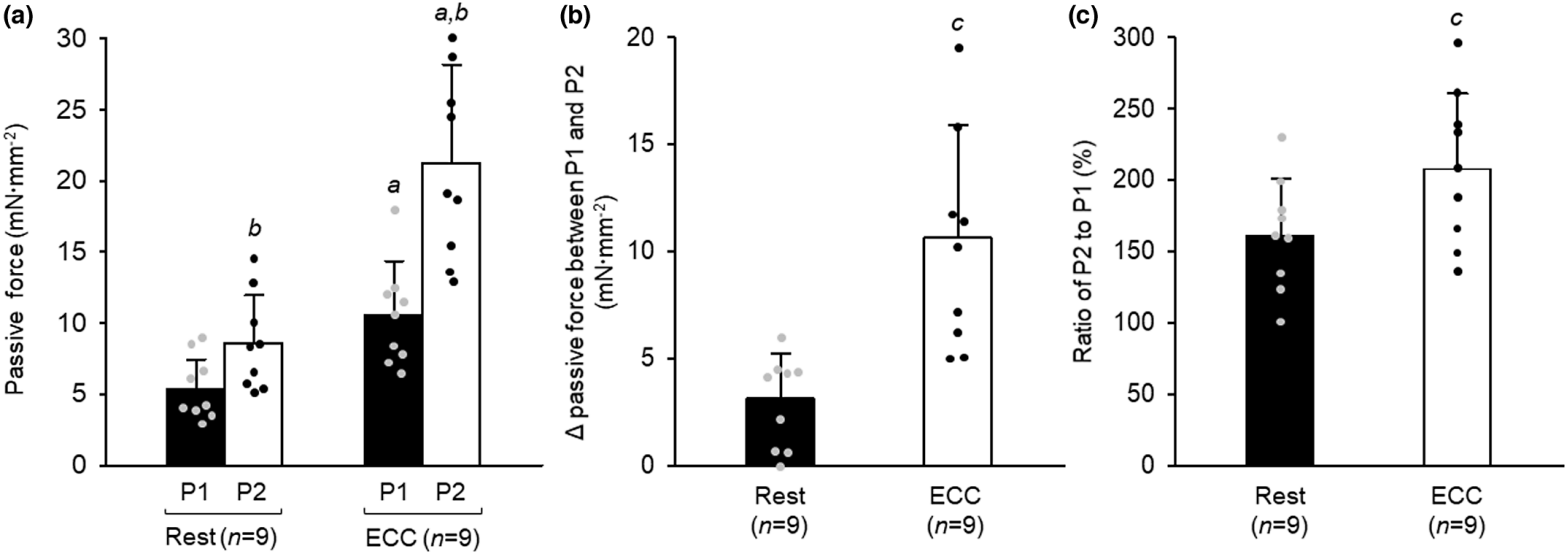
Effects of eccentric contraction and active stretch on passive force in skinned fiber. For the protocols of passive force measurement and eccentric contraction (ECC), see legend of Figures 1 and 2, respectively. (A) Passive force (P) 1 and 2. (B) Difference between P1 and P2. (C) Ratio of P2 to P1. Values are means + SD; *n* denotes number of fibers. Individual data are displayed as gray or black circles. ^*a*^ *p* <0.05, vs. rested fibers in matching condition (two‐way repeated ANOVA); ^*b*^ *p* <0.05, vs. P1 within fibers (two‐way repeated ANOVA); ^*c*^ *p* <0.05, vs. rested fibers (*t*‐test). Rest, rested fibers; ECC, fibers subjected to ECC.

A significant interaction between ECC and active stretch of SL was found for passive force (Figure 6A). ECC induced a 92.7% and 146.5% increase in P1 and P2, respectively. The active stretch of the SL produced an increase in passive force in both rested and ECC fibers. Both Δ passive force and a percent increase were greater in ECC than in rested fibers, indicating that ECC potentiates PFE (Figure 6B,C).

### 3.6 Ratio of passive force to maximal Ca^2+^‐activated force

**FIGURE 7:**
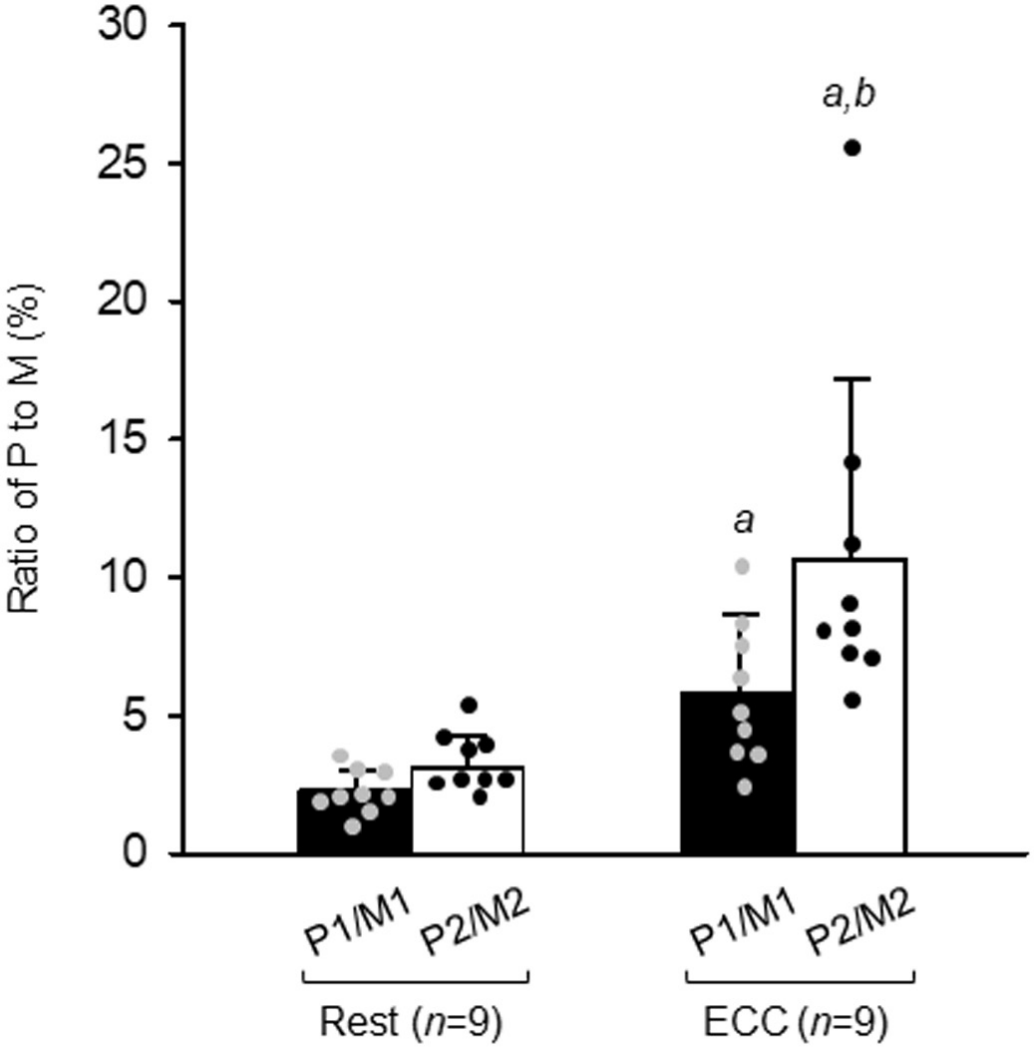
Effects of eccentric contraction and active stretch on ratio of passive force (P) to maximal Ca^2+^‐activated force (M) in skinned fiber. For the protocols of P and M measurement and eccentric contraction (ECC), see legend of Figures 1 and 2, respectively. Values are means + SD; *n* denotes number of fibers. Individual data are displayed as gray or black circles. ^*a*^ *p* <0.05, vs. rested fibers in matching condition (two‐way repeated ANOVA); ^*b*^ *p* <0.05, vs. P1/M1 within fibers (two‐way repeated ANOVA). Rest, rested fibers; ECC, fibers subjected to ECC.

To evaluate the contribution of the passive force to total force, the P/M ratio (P1/M1 and P2/M2) was calculated. There was a significant interaction between ECC and active stretch of the SL for these variables (Figure 7). ECC resulted in a significant increase in the ratio for both P1/M1 and P2/M2. A significant difference between P1/M1 and P2/M2 was also observed for ECC fibers.

## 4 DISCUSSION

In this study, we investigated the effects of ECC on FEs. Our previous study has indicated that ECC can induce an increase in passive force (Shi et al., 2022), which is confirmed by this study. The present study provides evidence, for the first time, that ECC leads to the potentiation of PFE but not RFE in skeletal muscle.

Joumaa et al. (2008) demonstrated that titin removal from the sarcomeres abolished PFE, indicating that titin is associated with FEs (Herzog, 2014a, 2014b). However, the precise mechanisms by which titin causes FEs remain unclear. Based on the results of the fluorescence study (DuVall et al., 2017), it has been proposed that, during active stretching of the myofibril, the proximal titin segments (the segment near the Z‐disk) in the I‐band region bind to actin and the binding causes a shortening of the free spring length of titin, resulting in stiffer titin and generation of greater force (Herzog, 2019). Another hypothesis that has been proposed is that the N2A domain binds to actin and the PEVK segment winds on the actin filament (Nishikawa et al., 2012). If these hypotheses were true, an increase in intrinsic titin stiffness would be expected to cause an increase in FEs. Our results of increased PFE and passive force in skinned fibers reinforce the above suggestion. The reason for the lack of changes in RFE is uncertain. As pointed out by Herzog and Leonard (2005), the mechanisms of RFE comprise two components: one related to the active contractile process and the other to the passive component. The negative effect of the active component could offset the positive effect of the passive component. Another possibility is that an increase in RFE was not detected because ECC induced only slight increase in passive force, which did not contribute to a significant increase in RFE.

In skeletal muscle, much greater force can be produced in ECC than in isometric and concentric contractions. In our experimental system, the max force developed by the rat GAS muscle is ∼2.5‐fold higher during ECC than during isometric contraction (results not shown). It is well known that exercise training that includes a substantial ECC induces a greater activation of the genes responsible for muscle remodeling than other types of training (Hody et al., 2019), owing to which the majority of competitive athletes employ high‐intensity eccentric training regimens. On the contrary, mechanical stress due to a stretch with ECC and a myofibrillar contraction leads to structural perturbations, such as triad deformation and/or sarcomere inhomogeneity (Kanzaki et al., 2022). Additionally, calpain‐induced degradation of proteins involved in excitation–contraction coupling occurs within a few minutes after the cessation of ECC (Zhang et al., 2008). These changes often result in large and long‐lasting force deficits in ECC muscle (Kanzaki et al., 2022).

It appears likely that P2 does not accurately reflect a passive force in M2, because Ca^2+^ activation slightly increases titin‐based stiffness (Labeit et al., 2003). However, given that P1, P2, M1, and M2 were assessed in the same manner for all samples, these data provide insight into the effect of ECC. Our results of an increase in the ratio (P1/M1 and P2/M2) of passive force to the max Ca^2+^ force indicate that passive force may contribute, to a greater extent, to total force output (passive force + active force) at a long SL in ECC than in rested muscle. Stimulation‐induced force in situ in ECC muscle was more depressed, compared with the max Ca^2+^ force in ECC fiber. The reduced force in ECC muscle will be ascribable to decreased Ca^2+^ release of the sarcoplasmic reticulum and impaired function of the myofibril, although the former is a predominant cause (Allen, 2001; Kanzaki et al., 2022; Watanabe, Aibara, & Wada, 2019). Increased passive force and potentiated PFE may function to attenuate ECC‐induced force reductions at the myofibrillar level.

In summary, we show ECC‐induced alterations in FEs in rat fast‐twitch skeletal muscle. PFE but not RFE is potentiated in ECC fibers, in which titin‐based passive force is increased. It appears likely that the PFE potentiation is due, at least in part, to the increased passive force. Considering that in human skeletal muscle, the max force is rarely used in a daily life, the present results on passive force increase, PFE potentiation, and low‐frequency fatigue in ECC fiber and muscle are of interest and importance. These results suggest that vigorous exercise including a substantial eccentric phase can enhance the contribution of passive force to daily used force output, although it is uncertain to what extent the present results obtained in rats under anesthesia can be extrapolated to exercising human muscles. It would be productive to investigate this issue as well as the mechanism(s) behind FEs in further studies.

## AUTHOR CONTRIBUTIONS

J.S. and M.W. conceived and designed the research; performed experiments; analyzed the data; interpreted the results of experiments; prepared the figures; drafted the manuscript; edited and revised manuscript; approved the final version of the manuscript.

## FUNDING INFORMATION

This study was supported by the Grants‐in‐Aid for Scientific Research of Japan (No. 20 K11335 and 23 K10714; M. Wada).

## CONFLICT OF INTEREST STATEMENT

The authors declare no conflict of interest.
